# Psychiatry meets neurodegeneration – A collaborative approach to dementia prevention^☆^

**DOI:** 10.1016/j.tjpad.2025.100317

**Published:** 2025-07-31

**Authors:** Carolin Kurz, Martin Haupt, Stefanie Auer, Nicola Lautenschlager, Alexander Kurz

**Affiliations:** a Department of Psychiatry and Psychotherapy, LMU University Hospital, LMU Munich, 80336 Munich, Germany; b Department of the History, Philosophy and Ethics of Medicine, Heinrich Heine University, Düsseldorf, Germany; c Teaching practice of the Clinic and Polyclinic for Psychiatry and Psychotherapy of the Heinrich Heine University, Düsseldorf, Germany; d Department for Dementia Research and Care Science, University for Continuing Education Krems, Austria; e Department of Psychiatry, The University of Melbourne, Melbourne, Victoria, Australia; f Royal Melbourne Hospital Mental Health Services, Royal Melbourne Hospital, Parkville, Victoria, Australia; g Department of Psychiatry and Psychotherapy, Centre for Cognitive Disorders, School of Medicine, Technical University of Munich, Munich, Germany

**Keywords:** Patient-centered care, Quality of life, Neurodegenerative diseases, Cognitive dysfunction/prevention & control, Dementia/prevention & control

## Abstract

The advent of amyloid-targeting therapies and biomarker-based risk stratification has transformed the understanding of Alzheimer’s disease and related disorders. These conditions are now recognized as chronic, detectable and modifiable, often presenting decades before clinical symptoms appear. While this paradigm shift enables earlier intervention, it also raises ethical and psychological challenges that necessitate a redefined role for psychiatry. Instead of merely supporting late-stage care, psychiatry is well-placed to facilitate risk communication, promote resilience, and encourage adaptive behavior in individuals navigating preclinical or prodromal neurodegeneration. This article outlines an ethical, stepwise communication framework, clarifies the distinction between diagnosis and probabilistic risk, and explores psychiatric contributions—from motivational models to lifestyle-based prevention—that bridge the gap between biological insight and subjective experience. By reinterpreting risk as a chance for intervention rather than resignation, psychiatry broadens the therapeutic scope and helps safeguard independence, dignity and quality of life—making it a pivotal participant in dementia prevention and individualized, person-centered care.

## 1 Introduction – from organic psychoses to precision psychiatry

Historically classified as ‘organic psychoses’, Alzheimer’s disease (AD) has held a unique position in psychiatry due to its clearly identifiable neuropathological features, which have profoundly shaped psychiatry’s emphasis on brain-based mechanisms in the understanding and treatment of mental illness [1]. With the advent of amyloid-targeting therapies and a shift towards a biologically based definition of Alzheimer's disease (AD), the historical arc comes full circle – linking past biological concepts to current approaches that emphasize pathology-driven interventions even before clinical symptoms emerge [2,3]. Psychiatry is faced with the task of not only interpreting pathological findings, but also contributing to prevention, emotional adaptation, and lifestyle change starting at the presymptomatic stage -empowering individuals to engage actively in their own brain health. Early diagnosis is becoming not only medically but also psychologically and ethically relevant, redefining psychiatry’s role from late-stage support to early intervention.

## 2 Conceptual foundations: bridging risk and diagnosis

Increased screening and recognition of neurodegeneration as a chronic disease with a long latency period - often 10 to 20 years - create valuable opportunities for early prevention, intervention, and informed decision-making [4]. The growing use of biomarkers in preclinical stages raises fundamental questions: when pathology is present without symptoms, is it disease or an at-risk state? [5,6]. Communicating probabilistic information demands care: without explanation, individuals may feel falsely reassured or needlessly alarmed. Ethical, value-based communication is essential to empower rather than to harm [7]. While the public health benefits of early detection are compelling, they must be weighed against ethical and practical challenges [8].

## 3 Presymptomatic screening – chance or risk?

Advances in biomarkers and therapies have sparked debate on how to define and communicate presymptomatic stages, highlighting tensions between identifying biological risk and avoiding premature diagnostic labels [5,6]. At the same time, defining early symptoms in neurodegenerative diseases remains a major challenge. Initial changes—such as apathy, anxiety, irritability, or subtle changes in personality - are often nuanced and gradual, and are increasingly conceptualized under the term *mild behavioral impairment* [9,10]. These symptoms can be difficult to distinguish from normal variation, adding complexity to early detection strategies. Nonetheless, biomarker-based approaches primarily target Alzheimer’s disease but frequently detect non-AD disorders - especially in ageing populations, where 30–50 % with early symptoms have alternative or mixed aetiologies [11]. Furthermore, a considerable number of people with preclinical AD may be excluded from amyloid-targeting therapies due to strict trial or treatment criteria [12,13]. These realities call for early identification not only as a treatment precursor but as a public health opportunity to provide prevention and reduce future burden [14]. The debate surrounding the early diagnosis of Alzheimer's disease (AD), particularly in the presymptomatic or at-risk stages, requires careful consideration of the potential individual benefits and risks, as well as integration into an evidence-based, ethically sound framework.

## 4 Psychiatry’s role in prevention of dementia

Earlier recognition opens a window for pro-active self-management—something long hindered by delayed diagnosis [15]. Given that 20–47 % of people over 60 express concern about developing dementia, many are motivated to take preventive action [16]. Mental health professionals offer unique expertise in communicating risk, managing uncertainty, and supporting emotional resilience—key to enabling behavior change and long-term motivation [[17], [18], [19]]. Psychiatry plays a direct role in reducing modifiable risk factors by offering targeted interventions that integrate psychosocial and somatic care. These include promoting healthy behaviors such as physical activity, smoking cessation, weight management, and controlling vascular risk factors like hypertension and hypercholesterolemia [[20], [21], [22]]. Additionally, the treatment of depression, anxiety, and sleep disturbances can further enhance cognitive outcomes and quality of life [23]. Despite methodological challenges—such as reliance on observational data, confounding variables, and heterogeneous study designs—prevention remains a cornerstone of dementia strategy. Early, structured, and inclusive interventions show great potential, particularly in underserved populations. To ensure effectiveness, it is crucial to investigate which modifiable risk factors, in which combinations and at which stages, yield the greatest benefit for specific dementia subtypes. Dementia prevention should be considered a collective societal responsibility, with psychiatry playing an integral role in fostering sustainable, interdisciplinary approaches.

## 5 Challenges and ethical considerations of early diagnosis

The growing focus on early detection must be balanced with consideration of the potential risks. Early diagnosis can cause anxiety and stigmatization, particularly since it is estimated that 20–30 % of individuals with positive AD biomarkers will never exhibit clinical symptoms [[24], [25], [26]]. Consequently, full-scale population screening is problematic and a targeted case-finding strategy - focusing on individuals with early symptoms or defined risk factors - offers a more effective, ethical, and resource-efficient approach – and also justifies initiating diagnostic steps [15,18,27].

**Fig. 1:**
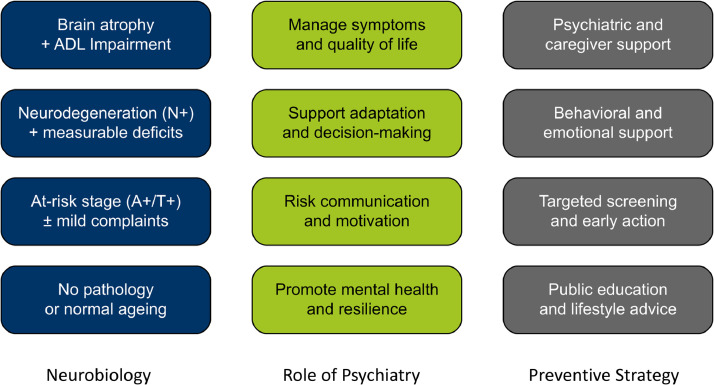
Navigating the Stages of Alzheimer’s Disease by a stepwise approach. Schematic overview of the clinical trajectory in Alzheimer’s disease, from normal ageing to dementia. The diagram integrates biological and clinical indicators with psychiatric roles and public health strategies. It distinguishes between at-risk and diagnostic stages and emphasizes proactive, ethically sound approaches to risk communication, prevention, and support. The model highlights the *psychiatric middle ground* - bridging biological changes and lived experience—thereby supporting person-centered care across all stages of the disease [5,6] ADL – activities of daily living.

The *psychiatric middle ground* offers a conceptual and clinical framework for navigating the presymptomatic and transitional stages of neurodegenerative diseases such as AD (see Fig. 1). It advocates for clear, ethically sound nomenclature that distinguishes between risk states, prodromal stages, and clinical diagnoses—ensuring transparent communication while minimizing harm [4,28,14].

## 6 Risk communication and behavior change

Psychiatry plays a key role in promoting sustained behavioral change by communicating risk, fostering motivation and resilience, and applying strategies such as self-regulation, motivation techniques, and habit formation to support long-term adherence to risk-reduction. Early identification through biomarkers can open a critical window for enabling individuals to adopt lifestyle changes that have a beneficial effect on brain plasticity and stress response [7,18,28,14,23,29].

## 7 From risk to resilience – A new preventive paradigm

Understanding why some individuals remain symptom-free despite biomarker-positive profiles points to resilience as a modifiable construct that can be actively promoted [[30], [31], [32], [33]]. Resilience factors such as optimism, emotional regulation, social connectedness, structured routines, and relationship building carry biological significance and may mitigate the impact of progressive neurobiological alterations [30]. Effective dementia prevention requires more than awareness - it depends on enabling and sustaining meaningful behavior change across individual and societal levels. Key principles include the promotion of acceptance, the equipping of individuals with practical skills and the encouragement of a mindset that is adaptive rather than merely reactive [34].

## 8 Shifting the focus toward quality of life and personhood

Independent of eligibility for amyloid-targeting therapies, treatment should prioritize factors that affect quality of life rather than focusing solely on cognitive decline. Effective medical treatment goes beyond pathology and should include supporting functional abilities, compensating for difficulties, improving communication and promoting overall well-being while maintaining hope, self-determination and a sense of purpose to help individuals adapt to their changing circumstances and counters age-related stereotypes [[35], [36], [37]]. Recognizing the potential of individuals at every stage of life is essential to fostering a culture of respect, equity and inclusion in health care [38]. Tailoring therapies to cognitive and emotional changes, engaging caregivers as partners in care, and providing structured, accessible interventions enhance equitable and effective treatment [39,40].

## 9 Expanding psychiatric impact through public health strategies

To shift dementia care toward prevention, early diagnostics must be embedded in public health infrastructures across diverse populations and settings. Access to healthcare via community-based, low-threshold formal resources such as pharmacies, or via telehealth, should be promototed [41,42]. Task shifting within multidisciplinary teams allows nurses and community health workers to conduct screening, monitoring, and education, easing pressure on specialists. Psychiatry plays a central role: equipping individuals with coping skills, training professionals in ethical communication, and embedding mental health into dementia outreach, but requires long-term investment in decentralized infrastructure.

## 10 Addressing gaps in early detection and communication

Psychiatry, as part of a preventive approach to dementia, must evolve - and has much to offer. It fills a critical gap in dementia care by contributing structured, ethically grounded frameworks for early identification and communication. Psychiatry ensures that information is conveyed clearly, culturally sensitively, and in an empowering way—helping vulnerable individuals navigate uncertainty and engage in proactive health decisions [14]. When at-risk identification is framed not as a deterministic outcome but as an opportunity for action, individuals are more likely to assume responsibility, pursue personalized preventive steps, and potentially influence the course of disease [7]. As cognitive decline affects multiple dimensions of life, including identity, relationships and emotions, an individualized approach that emphasizes dignity, strengths and individual history is required [43]. This approach goes beyond symptom management and focuses on preserving individuality and incorporating coping strategies [[43], [44], [45], [46]].

## 11 Conclusion

In AD and related disorders the focus has shifted are from being defined primarily by dementia as an end-stage symptom of brain pathology towards maintaining independence, quality of life, and participation. By aligning biological insights with psychological adaptation, psychiatry bridges the gap between pathology and personhood, where psychiatry meets neurodegeneration.

**Table:** 

**Key Contributions and Addressed Gaps in Dementia Prevention** • **Procative role of psychiatry:** Repositions psychiatry from late-stage support to an early, preventive role in neurodegeneration – addressing risk communication, emotional adaptation, and lifestyle guidance. • **Bridging biological and psychosocial models:** Connects biomarker-based diagnostics with behavioral and psychological interventions • **Clarification of diagnosis vs. at-risk:** Frames biomarker findings not as deterministic diagnoses, but as modifiable risks—highlighting the need for psychiatric support in communicating uncertainty. • **Risk factors as targets for intervention:** Emphasizes modifiable risk factors positioning psychiatry to address mental health-related risks across the life course that influence brain health. • **Resilience as an intervention target:** Redefines resilience not just as an individual trait but as a biopsychosocial resource that can be promoted through psychiatric techniques. • **Concrete behavioral frameworks:** Introduces evidence-based behavior change models into dementia prevention—an area often overlooked. • **Focus on quality of life:** Shifts attention from pathology to personhood—emphasizing autonomy, and inclusion across all stages of cognitive decline. • **Public health relevance:** Proposes scalable, low-threshold, and ethically sound approaches that extend psychiatric impact beyond specialty clinics. • **Ethical communication in early detection:** Promotes clear, value-sensitive communication of probabilistic risk—empowering individuals to take preventive action without inducing unnecessary distress.

## Statements relating to our ethics and integrity policies

This manuscript is a personal perspective and does not involve original research, clinical trials, patient data, or external funding. As such, no data availability statement, funding statement, conflict of interest disclosure, ethics approval statement, or patient consent statement is applicable. Additionally, there is no material reproduced from other sources that would require permission, nor is this work associated with any clinical trial registration. The authors affirm that there are no ethical concerns related to this manuscript and no conflicts of interest to disclose.

## CRediT authorship contribution statement

**Carolin Kurz:** Writing – review & editing, Writing – original draft, Conceptualization. **Martin Haupt:** Writing – review & editing, Writing – original draft, Conceptualization. **Stefanie Auer:** Writing – review & editing, Writing – original draft, Conceptualization. **Nicola Lautenschlager:** Writing – review & editing, Writing – original draft, Conceptualization. **Alexander Kurz:** Writing – original draft, Conceptualization.

## Declaration of competing interest

The authors declare that they have no known competing financial interests or personal relationships that could have appeared to influence the work reported in this paper.
